# Sub-optimal breastfeeding of infants during the first six months and associated factors in rural communities of Jimma Arjo Woreda, Southwest Ethiopia

**DOI:** 10.1186/1471-2458-12-363

**Published:** 2012-05-18

**Authors:** Dessalegn Tamiru, Tefera Belachew, Eskindir Loha, Shikur Mohammed

**Affiliations:** 1 Department of Public Health, Arbaminch University, Arbaminch, Ethiopia; 2 Department of Population and family Health, Jimma University, Jimma, Ethiopia; 3 Department of Public Health, Hawassa University, Hawassa, Ethiopia

## Abstract

**Background:**

Studies have shown that sub-optimal breastfeeding is major contributor to infant and young child mortality in Ethiopia. To address this problem, infant and young child feeding guideline was developed in 2004 and interventions have been going on based on the guidelines. There is no study that assessed whether the infant and child feeding practices are according the guideline or not. This study was carried out to assess sub-optimal breastfeeding practices and associated factors among infants from birth to six months in rural communities of Jimma Arjo Woreda in the Southwest Ethiopia.

**Methods:**

A cross-sectional study was carried out from December to January 2009. Quantitative data were collected from a sample of 382 respondents supplemented by qualitative data generated using in-depth interviews of 15 index mothers. Multivariable logistic regression model was used to identify predictors of timely initiation of breast feeding and non-exclusive breast feeding among mother-infant pairs.

**Results:**

More than three fourth of mothers breastfeed their infants sub-optimally. Thirty-seven percent of mothers initiated breastfeeding later than one hour after delivery, which was significantly associated with not attending formal education (AOR = 1.05[95%CI: 1.03, 1.94]) and painful breastfeeding experiences (AOR = 5.02[95%CI: 1.01, 10.08]). The majority (67.02%) of mothers had no knowledge about exclusive breastfeeding. Non-exclusive breastfeeding was negatively associated with child’s age of 0-2 months (AOR: 0.27[95%CI: 0.16, 0.47) and 3-4 months (AOR = 0.43 [95%CI: 0.25, 0.73) and ownership of radio (AOR = 0.56[95%CI: 0.37, 0.88]), but positively associated with the practice of discarding colostrums (AOR = 1.78[95%CI: 1.09, 4.94]).

**Conclusion:**

The findings showed that the majority of mothers sub-optimally breastfeed their children in the study area. As most of the mothers do not have knowledge on the exclusive breast feeding. Enhancing community based behavior change communications using multiple channels including radio and folk media is recommended to reduce sub-optimal breast feeding practices and associated consequences among children in the study area.

## Background

Breastfeeding renders many health and developmental advantages through promoting and protecting maternal and child health. Optimal breastfeeding is one of the most effective preventive health measures against diarrheal diseases and child mortality. Studies indicate that breast milk protects infants from infectious and chronic diseases [1,2].

Globally, 60% of the infant and young child deaths occur due to inappropriate infant feeding practices and infectious disease from which two-thirds of these deaths are attributable to sub-optimal breastfeeding practices [2]. Inappropriate infant feeding practices could have negative effect on child growth and development, especially in developing countries where accessibility of basic health service is not sufficient [3-5]. In Ethiopia, malnutrition associated with poverty, household food insecurity and low access to health care is reported to be responsible for about 57% of all under five deaths [4,6]. Malnourished children who survive are more frequently sick and suffer the life-long consequences of impaired development [5]. Infant feeding is a complex issue that has implications not only on infant’s nutritional and health status, but also on his/her psychological development and the development of proper eating habits [2,7,8].

According to WHO, exclusive breastfeeding is defined as the practice of feeding only breast milk (including expressed breast milk) and no other liquids or solids with the exception of drops or syrups consisting of vitamins, mineral supplements or medicine [2,4]. Globally, there are complex arrays of socio*-*cultural influences related to acceptable infant feeding practices [2,3,9]. The gap between breastfeeding practices and recommendations is increasing in developing countries where socio-economic and traditional practices have considerable implications on breastfeeding and child feeding practices [3,10,11]. Early and abrupt cessation of breastfeeding, followed by an introduction of dirty, unsound artificial feeding of infants with very dilute milk products is common. As a result, infants and young children are more vulnerable to infection with different pathogens since their body nutrient store is not well developed [3,4,8].

Studies indicated that traditional and cultural beliefs related to breastfeeding have negative influence on the practice of exclusive breastfeeding in developing countries [9,12,13]. In some societal cultures, plain water, butter and other foods are given for newborn infants, while colostrum is discarded as unclean. Breastfeeding is started when the breast milk becomes clearer after a few days. It is considered as preventive measures against disease and symbol of welcoming the baby [9,11,12]. Failure to exclusively breastfeed young infants and the introduction of liquid and solid foods at too early age increases the risk of diarrheal disease and mortality in Africa [3,14]. Globally, less than 35% of mothers exclusively breastfeed their infants during the first few months of life and the problem is highly increasing in sub-Saharan countries [2,15]. Studies indicated that in the developing world 39% of mothers exclusively breastfeed their infants up to 6 months; while in some countries no mother exclusively breastfeeds to 6 months [16,17].

According to the 2005 Ethiopian Demographic and Health Survey (EDHS 2005), 96% of children in Ethiopia, both urban and rural, have ever been breastfed during some period in their lives; however, breastfeeding is not optimal [4]. Nationally, 69.1% of newborns are put on breast within one hour of birth and less than 80% of infants 2 months old are exclusive breastfed. However, this proportion rapidly drops to 38% at the age of 6 months [4,6]. The proportion of ever breastfed children ranged from a low of 93% in Addis Ababa to a high of 99% in Harari [4]. In Oromia region, according to EDHS 2005, 26% of infants were given pre-lacteal feed and only 45.8% of infants received colostrum [5]. Although breastfeeding is one of the components of Primary Health Care in Ethiopia, a wide range of harmful infant feeding practices are documented even after implementations of infant and young child feeding recommendations in Ethiopia. However, there are no studies which documented breastfeeding patterns and factors associated with sub-optimal breastfeeding practices in the study area. Therefore, the objective of this study is to assess breastfeeding patterns and identify factors associated with sub-optimal breastfeeding practices among infants during the first 6 months in rural communities of Jimma Arjo Woreda.

## Methods and materials

### Study setting and sample

A cross-sectional community based study was carried out in Jimma Arjo Woreda, Oromia region, South West of Ethiopia, located at 327 km from Addis Ababa. From 20 rural kebeles(small villages), 8 were randomly selected using probability proportional to size allocation method. Then, mothers-infant pairs were randomly selected. A total of 384 mothers of index children aged 0 to 6 months were interviewed from December to January 2009. Fifteen key informants were identified purposively for in-depth interview on sub-optimal breastfeeding practices. The sample size was calculated using a formula for estimation of single proportion as follows:

(1) $$\text{n}=\frac{{\left(\text{Z}\alpha /2\right)}^{2}\text{p }\left(1-\text{p}\right)}{{\text{d}}^{2}}$$

Where

Z = Standard normal variable at 95% confidence level (1.96)

d = Margin of error (0.05)

P = Expected prevalence of non-exclusive breastfeeding (50%).

This study included mothers who are permanent residents of the selected kebeles and those who had infants aged from 0 to 6 months; however, HIV positive mothers were not included.

### Measurements

The quantitative data were collected using structured questionnaires, while the in-depth interview and observation of infant positioning and attachment during breastfeeding were done using structured guide and an observation checklist, respectively. A structured questionnaire was used to collect data on the socio-demographic characteristics, maternal and child characteristics, knowledge on breastfeeding and cultural beliefs regarding infant feeding practices. In addition, in-depth interview guide was used to generate descriptions of women’s knowledge, experiences and perceptions related to infant feeding. This allowed each participant to contribute her own knowledge of breastfeeding within broadly defined research themes. The main target of the in-depth interview was to capture mothers’ first-hand knowledge about a topic of interest. All interviews were conducted by the data collectors in the participants’ homes. Both questionnaire and interview guides were prepared in English, translated to Afan Oromo and finally retranslated to English language. The questionnaire was pre-tested before the actual data collection. Additional modifications were made to the questionnaire in terms of in terminologies and formatting based on the pretest findings. The supervisors checked each completed questionnaire and principal investigator monitored the overall quality of the data collection.

In this study, optimal breastfeeding was defined as adherence to standard recommendations such as initiation of breastfeeding within one hour, giving colostrums, exclusive breastfeeding for 6 months and introduction of safe, nutritious and age appropriate complementary food around 6 months and good breastfeeding skills. Delayed initiation of breastfeeding was defined as not starting breastfeeding within one hour after delivery.

Exclusive breastfeeding was defined as not giving and liquid, semisolid or solid food apart from breast milk with the exception of drops or syrups consisting of vitamins, mineral supplements or medicines.

### Statistical analysis

The data were entered in double, checked for missing values and outliers, and analyzed using SPSS (SPSS Inc. version 15.0, Chicago, Illinois). First, bivariate analyses were carried out to identify candidate variables for the multivariable model. Second, to identify the predictors of suboptimal breastfeeding, only variables that were significantly associated on bivariate analyses were entered in the multivariable logistic regression model with delayed initiation of breastfeeding as dependent variable. Third, we fitted a second multivariable logistic regression model to identify factors that predict non-exclusive breastfeeding. All tests were two-sided and P < 0.05 was considered statistically significant. We report the results as adjusted odd ratios (AOR) and 95% confidence intervals.

Breastfeeding observation was evaluated based on the criteria set by national breastfeeding recommendations. A child is well positioned if infant body and head is straight; head and body facing breast and well supported with skin-to-skin contact and if the infant’s whole body is supported. If one of the criteria is not fulfilled it was considered as poor positioning. A child is well attached if chin of an infant touched breast and lip opened widely; lower lip turned down ward with areola is more visible above than below. If one of the criteria was not fulfilled it was considered as poor attachment.

The qualitative data were analyzed using an open coding system which involved note taking, coding, sorting, examining, comparing and categorizing data and writing the findings. On the completion of the discussion, participants were invited to provide comments on final narrative. Finally, these categories of data were presented in narrative in triangulation with the quantitative results using well-said verbatim of the study participants as illustrations.

The study was financially supported by Hawassa University College of Medicine and Health Science. Informed verbal consent was obtained from all the study participants and permission was also obtained from the local administrative units.

## Results

Out of 384 participants intended to be included, a complete response was obtained from 382(99.5%) respondents. The mean age of mothers was 25.9 years (±5.15) the range being from 15 to 40 years. Majority of mothers 375(98.2%) were married (Table 1).

**Table 1:** Socio-demographic Characteristics of Respondents in Jimma Arjo Woreda, 2009

**Variable**	**Frequency**	**Percent**
Family size (n = 382)*		
2-5	241	63.1
>5	141	36.9
Current marital status (n = 382)		
Married	375	98.2
Unmarried	7	1.8
Maternal age (in years)		
15-20	43	11.3
21-25	191	50
26-30	98	25.6
31-35	40	10.4
>35	10	2.7
Mothers’ education (n = 382)		
Illiterate	274	71.7
Can read and write	45	11.8
Primary education	57	14.9
Secondary and above	6	1.6
Ethnicity (n = 382)		
Oromo	380	99.5
Amhara	2	0.5
Religion (n = 382)		
Protestant	243	63.6
Orthodox	136	35.6
Muslim	3	0.8
Infant age		
<1 month	19	5
1-2 months	103	27
3-4 months	89	23.3
5-6 months	171	44.7
Taking health education(n = 382)		
Yes	168	43.98
No	214	56.02
Delivery type		
Caesarean section	8	2.1
Vaginal delivery	374	97.9
Owner of radio (n = 382)		
Yes	181	47.4
No	201	52.6
Antenatal Care visit		
Yes	190	49.7
No	192	50.3
Delivery assistant		
Traditional Birth Attendants	311	81.4
Health Workers	53	13.9
Others^†^	18	4.7

*Mean ± SD = 5.21 ± 1.80.

^†^Relatives, health extension workers.

Only 24.6% of mothers breastfed their infants optimally. Among those who ever breastfed, more than half of mothers (62.6%) initiated breastfeeding within first hour of delivery and 272 (72.5%) gave colostrum to their infants, and 43.3% of mothers provided butter and 53.2% gave rue(“*tena-addam”)* to infants to protect them against stomachache and common cold, respectively. One hundred eighty three (47.91%) mothers reported to have exclusively breastfed. The proportion of exclusive breastfeeding was 37.9% at the end of the first month which dropped to 9.9% at age of 6 months. More than half of mothers (57.22%) breastfed 8 or more times per day and 66.21%) and 59.9% of infants were poorly positioned and attached during breastfeeding, respectively (Table 2).

**Table 2:** Patterns of breastfeeding experiences in Jimma Arjo Woreda, 2009

**Variables**	**Frequency**	**Percent**
Ever breastfed (n = 382)		
Yes	375	98.17
No	7	1.83
Initiation of breastfeeding (n = 375)		
Within one hour	235	62.6
After one hour	140	37.4
Giving colostrum (n = 375)		
Yes	272	72.5
No	103	27.5
Pre-lacteal feeding (n = 30)		
Butter	13	43.3
Rue *(“Tena-addam”)*	16	53.3
Cow’s milk	1	3.4
Currently breastfeeding (n = 382)		
Yes	367	96.07
No	15	3.93
Frequency of breastfeeding (n = 367)		
≥8	210	57.22
< 8	157	42.78
Breastfeeding at night (n = 367)		
Yes	348	94.82
No	19	5.18
Currently Exclusive Breastfeeding (n = 382)		
Yes	183	47.91
No	199	52.09
Exclusive Breastfeeding by month (n = 382)		
0-1	145	37.9
2-3	118	30.9
4-5	81	21.3
6	36	9.9
Method of giving fluids/foods (n = 200)		
Bottle	48	24.13
Spoon	79	39.69
Others^†^	72	36.18
Child positioning (n = 367)		
Good positioning	124	33.79
Poor positioning	243	66.21
Child attachment (n = 367)		
Not at all attached	15	4.08
Poorly attached	220	59.95
Well attached	132	35.97
Breastfeeding(n = 382)		
Optimal	94	24.6
Sub-optimal	288	75.4

^†^cup, hands.

Almost all mothers (99.7%) did not believe that breastfeeding takes too much time. Eleven (2.87%) mothers reported that breastfeeding was painful and 12 (3.2%) mothers said their breast was too small to breastfeed. One-hundred twenty-three (32.8%) mothers had information about exclusive breastfeeding from 5-6 months. Few mothers state that colostrum is unclean (10.93%) and it causes a disease (5.6%), Table 3.

**Table 3:** Mother’s knowledge and attitude on breastfeeding in Jimma Arjo Woreda, 2009

**Knowledge and attitude**	**Frequency**	**Percent**
**Knowledge**		
Have information of EBF to 5-6 months	123	32.8
Know the Importance Colostrum	7	1.93
**Attitude**		
Believe breastfeeding takes time	1	0.3
Believe formula feeding is cheap	10	2.63
Feel breastfeeding is painful	11	2.87
Believe small breast cannot produce enough milk	12	3.2
Believe colostrum is bad	41	10.93
Believe colostrum causes illness	21	5.6

* Percentages do not add up to 100 as there was more than one response.

* EBF = Exclusive Breastfeeding.

Though discarding colostrum is not significantly associated with socio-demographic variables, delayed initiation of breastfeeding was positively associated with nipple pain and lack of formal education. Multivariable regression model showed that mothers who had no education were 5% more likely to initiate breastfeeding after 1 h of child birth than those who attended formal education (AOR = 1.05[95%CI: 1.03, 1.94]). Similarly, mothers who had nipple pain were 5 times more likely to have delayed initiation of breastfeeding (AOR = 5.02[95%CI: 1.01, 10.08]) (Table 4).

**Table 4:** Odds ratios and 95% confidence intervals from bivariate and multivariable logistic regression model predicting the likelihood that mothers in Jimma Arjo woreda having delayed initiation of breastfeeding, 2009

**Delayed Initiation of breastfeeding**				
**Predictors**	**Yes**	**No**	**Crude OR(95%CI)**	**Adjusted OR(95%CI)**
	**No. (%)**	**No. (%)**		
Maternal education				
Illiterate	106(39.3)	164(60.7)	2.051.09, 3.84)*	1.05 (1.03,1.94)*
Can read and write	16 (37.2)	27 (62.8)	2.13(0.93, 4.09)	0.51(0.22, 1.18)
Primary school and above	18(29)	44 (71)	1	1
Owner of radio	67 (37.9)	110(62.1)	0.97(0.64, 1.47)	__
Yes				
No				
	73 (36.87)	125 (63.13)	1	
Antenatal care follow up				
Yes	69(37.5)	115 (62.5)	0.94(0.62, 1.43)	__
No	71 (37.1)	120 (62.9)	1	
Marital status				
Married	137(37.2)	231(62.8)	0.67(0.13, 3.59)	__
Others	3 (42.9)	4(57.1)	1	
Taking HE about FP				
Yes	66 (39.3)	102(60.7)	1.2(0.79, 1.7)	
No	74(36.81)	133 (63.19)	1	
Delivery assistant				
Traditional birth attendants	115(37.22)	194(62.78)	1.72(0.54, 5.45)	
Health Extension Workers	10 (40)	15 (60)	2.44(0.63, 9.38)	__
Nurse	11 (44)	14 (66)	1.80(0.44, 7.31)	
Relative	4 (25)	12 (75)	1	

Significant at p < 0 .05, Adjusted OR = Adjusted Odd Ratio, Crude OR = Crude Odd Ratio.

HE = Health education, FP = Family Planning.

Similarly, the qualitative data indicated that all mothers did not know when breastfeeding should be initiated after delivery, but seven mothers reported that they initiated breastfeeding within one hour of child birth. For instance 22 years old merchant mother said:

"“…Actually I started breastfeeding within one hour after delivery. My mother helped me to start breastfeeding.”"

The majority of mothers reported that they did not remember giving breast milk as they were busy with different activities including cutting of umbilical cord and washing the blood. For instance 29 years two farmer mothers said:

"“…I started breastfeeding after everything was settled as there was nobody with me to help me give breast milk for the child”"

In addition, two mothers reported that they delayed to initiate breastfeeding because of their previous nipple pain during breastfeeding.

Non-exclusive breastfeeding was positively associated with child’s age. Mothers who had 0-2 months old infants were 73% less likely to non-exclusively breastfeed than those who had 5-6 months old infants (AOR = 0.27[95%CI: 0.16, 0.47). Similarly, mothers who had 3-4 months old infants were 57% less likely to non-exclusively breastfeed than those who had 5-6 months old infants (AOR = 0.43 [95%CI:0.25, 0.73) (Table 5).

**Table 5:** Odds ratios and 95% confidence intervals from bivariate and multivariable logistic regression model predicting the likelihood that mothers in Jimma Arjo Woreda not exclusively breastfed during the first 6 months, 2009

**Predictors**	**Non-exclusive breastfeeding**			
	**Yes**	**No**	**Crude OR (95%CI)**	**Adjusted OR(95%CI)**
	**No. (%)**	**No. (%)**		
Mothers age				
15-20	18 (41.9)	25 (58.1)	0.48 (0. 12, 1.95)	
21-25	97 (50.8)	94(49.2)	0.69 (0.19, 2.51)	_
26-30	62 (63.3)	36(36.7)	1.45 (0.30, 4.34)	
31-35	16 (40)	24 (60)	0.44(0.11, 1.83)	
36 and above	6 (60)	4 (40)	1	
Child’s age				
0-2	44(36.1)	78(63.9)	0.28(0.17, 0.46)**	0.27(0.16, 0.47)**
3-4	41(46.1)	48(53.9)	0.42(0.25, 0.70)**	0.43(0.25, 0.73)**
5-6	114(66.7)	57(33.3)	1	1
Fetching water				
Mother	161(53.3)	141(46.7)	0.79(0.48, 1.30)	__
Children/husband	38 (47.5)	42 (52.5)	1	
Ownership of radio				
Yes	104(57.5)	77 (42.5)	0.66(0.44, 0.99)*	0.56(0.37,0.88)*
No	95(47.3)	106(52.7)	1	1
Antenatal Care follow up				
Yes	103(52.1)	87 (45.8)	0.85(0.57, 1.26)	__
No	96 (50)	96 (50)	1	
Delivery assistant				
Traditional birth attendants	164(52.7)	147(47.3)	0.86(0.31, 2.36)	0.67(0.22,1.96)
Health Extension Workers	16 (55.2)	13 (44.8)	0.96(0.28, 0.97)*	0.88(0.23,3.39)
Nurse	10 (41.7)	14 (58.3)	0.56(0.16, 1.10)	0.38(0.11,1.50)
Relative	9 (50)	9 (50)	1	1
Discarded colostrum				
Yes	42 (40.8)	61 (59.2)	1.87(1.18, 2.96)**	1.78(1.09,4.94)**
No	157(56.3)	122(43.7)	1	1
EBF Knowledge				
Yes	57 (48.6)	66 (51.4)	1.30(0.87, 1.94)	__
No	142(36.1)	117(63.90)	1	

EBF = Exclusive Breastfeeding.

There was also a negative association between ownership of radio and non-exclusive breastfeeding practices. Mothers (households) who had radio were 44% less likely non-exclusively breastfeed compared to those who had no radio (AOR = 0.56[95%CI: 0.37, 0.88]). It was also observed that mothers who discarded colostrum were nearly twice more likely to nonexclusively breastfeed (AOR = 1.78[95%CI: 1.09, 4.94]) (Table 5).

In-depth interview of mothers about exclusive breastfeeding, five mothers stated that breast milk is sufficient for infants up to 4 months. For instance 25 years Traditional Birth Attendant mother said:

"“Before I gave birth, I had planned to give only breast milk for 4 months. I will not provide an additional food until my child can sit with hand support…”"

Some mothers provided cow’s milk and soup to infants after a few days of birth. Twenty three years old mothers said:

"“…The baby started to open his mouth, when somebody is eating food since he is not satisfied with breast milk.”"

## Discussion

Breastfeeding was a common practice and considered as natural gift in rural communities of Jimma Arjo Woreda. However, this study showed that 75.4% of mothers in the study area practice sub-optimal breastfeeding. This might be due to lack of knowledge about optimal breastfeeding practices. The fact that large proportion of mothers practiced sub-optimal breastfeeding after five years of development of the national infant and young child feeding guideline indicates the need for strengthening behavior change communication on optimal infant and young child feeding practices.

The mothers were asked when they initiated breastfeeding and 62.6% of mothers reported that they initiated breastfeeding within one hour of infant birth, which is relatively low when compared with 69.1% reported by EDHS 2005 [6]. However, it was much higher than 9.9% reported from in Turkey [18]. Delayed initiation of breastfeeding was more common among mothers who had no education compared with mothers who attended formal education which was similar with the findings from Turkey [18]. This might be due to the influences of cultural and traditional malpractices influencing the practice of initiating breastfeeding negatively and lack of knowledge about the importance of initiating breastfeeding within one hour. In addition this studies also indicated that mothers who had nipple pain were delayed to initiate breastfeeding within one hour delivery. Findings from Uganda also indicated that cracked nipples, breast engorgement and mastitis are some constraints of optimal breastfeeding practices [17]. Direct interview with mothers also showed that some mothers delayed to initiate breastfeeding due to their previous nipple pain which is consistent with the report from United State of America [19].

Despite national attempts to disseminate the importance of exclusive breastfeeding, more than half (52.1%) of mothers in the rural communities of Jimma Arjo Woreda did not exclusively breastfeed for the first six months, which is relatively high compared to the report of EDHS 2005[6]. As infants grew older, the proportion of exclusive breastfeeding progressed to lower values indicating the overall lower duration of exclusive breastfeeding in the study community. This is common in many developing countries as majority of mothers believe that breast milk is not sufficient for infants up to 6 months and traditionally give water and butter to new born [11,12,16]. Our findings indicated the older the child, the more likely that he/she is non-exclusive breastfed which is consistent with the report at national level[20]. The majority of mothers (67.2%) had poor knowledge about the duration of exclusive breastfeeding. The qualitative data also showed that some mothers want to exclusively breastfed up to 4 months, but majority of study participants did not agree with this idea. Studies from Tanzania and Nigeria also showed that mothers gave pre-lacteal feeds due to their belief that it prevent against disease [10-12].

This study indicated that availability of radio had significant contribution in promoting optimal breastfeeding practices. Mothers those who have radio were less likely to non-exclusively breastfeed compared to those who did not have radio. This could be due to the nutrition education messages given through the radio. Study conducted in Bolivia and Tanzania also showed that television and radio ownership had significant role in increasing mothers’ knowledge on optimal breastfeeding practices [10,13].

Traditional malpractices like pre-lacteal feeding and early introduction of complementary food is common barriers to optimal breastfeeding practices. Pre-lacteal feeding inhibits giving colostrum as mothers give pre-lacteal feeds to replace the colostrum they discard. By the same token, findings from this study indicated that mothers who discarded colostrum were more likely to non-exclusively breastfeed than those who gave colostrum for their children. Similar findings from Gambia and Turkey also showed additional food and some fluids were provided instead of colostrum which they discarded [12,18].

This study also indicated that children were given complementary food before 6 months, which is common both in developing and developed countries [2,3,16]. Most of the mothers provided foods like cow’s milk and soup as they thought that breast milk was insufficient. This finding was consistent with study done in South Africa in which babies were breastfed to stop them crying, to quench their thirst or to put them to sleep. An additional food is usually given early when mothers think that breastfeeding alone does not satisfy the child [9].

In contrast to national and global recommendations, more than one-third (27.5%) of mothers discarded colostrum considering that it is water and causes disease. National studies indicated that 38% of mothers did not give colostrum for new born infants [21]. This figure is relatively high compared to the present study. The qualitative data showed that mothers who had information about the importance of colostrum did not discard it although they did not attend formal education. Studies showed better knowledge about breastfeeding is the most important factors for giving colostrum and exclusively breastfeed up to 6 months [9,18].

Infant positioning and attachment during breastfeeding were not properly practiced in this community compared to national and global standard recommendations. For an effective breastfeeding, an infant should be well attached and positioned during breastfeeding [22]. However, in our study more than half of mothers (66.21%) poorly positioned their infants and 59.7% of the infants were poorly attached during breastfeeding. There was no significant association between breastfeeding skills and maternal and infant socio-demographic characteristics.

The findings of this study can have significant implication for national nutrition strategy in the promotion of optimal infant feeding practices and for achievement of millennium development goals for reduction of child mortality. However, there are some limitations. The cross-sectional nature of this study limited drawing causal inferences from the association between the factors and sub-optimal breastfeeding practices. There might be recall bias on breastfeeding practices as the mothers could forget when they introduced an additional food, but by relating the practices with local events and training the interviewers how to probe the mother to remember what she gave to her child, the recall bias was minimized. There might be also reporting bias during face-to-face interviews which was tried to be minimized by selecting data collectors from each kebele.

## Conclusions

The findings showed that there is high prevalence of sub-optimal breastfeeding (75.4%) in the study area. Sub-optimal breastfeeding practice such as delayed initiation of breastfeeding was positively associated with mother’s prior experience of nipple pain and lack of formal education. Exclusive breastfeeding was highly affected by cultural malpractices. Infants’ age, ownership of radio and discarding colostrum were the most important predictors of non-exclusive breastfeeding in the study area. Some mothers had a negative attitude towards giving colostrum to their babies. More than half of the mothers practiced poor positioning and attachment of their infants during breastfeeding which is contrary to the global and national recommendations. Strong community based behavior change communication on the importance of optimal breastfeeding practices is recommended to be instituted using Health Extension Workers and local community’s resource people as key actors to change the suboptimal breast feeding practices in the study community.

## Competing interests

The authors declare that they have no competing interests.

## Authors’ contributions

The authors’ responsibilities were as follows: DT, TB, EL, SM: Designed and supervised the study and ensured quality of the data and made a substantial contribution to the local implementation of the study and assisted in the analysis and interpretation of the data. DT did the analysis and drafted the paper had the primary responsibility for the content. TB the corresponding author submitted the paper for publication. We are extremely grateful to mothers involved in the study. All authors critically reviewed the manuscript. All authors read and approved the final manuscript.

## Pre-publication history

The pre-publication history for this paper can be accessed here:

http://www.biomedcentral.com/1471-2458/12/363/prepub
